# Increase in dengue fever in travellers returning from Egypt, Germany 2023

**DOI:** 10.2807/1560-7917.ES.2024.29.5.2400042

**Published:** 2024-02-01

**Authors:** Christina Frank, Raskit Lachmann, Hendrik Wilking, Klaus Stark

**Affiliations:** 1 Department for Infectious Disease Epidemiology, Robert Koch Institute, Berlin, Germany

**Keywords:** Dengue, Egypt, Aedes aegypti, Hurghada, tourism, travel, infection

## Abstract

Dengue virus (DENV) infections after exposure in Egypt increased in Germany in 2023, with 36 cases vs zero to eight in 2017 to 2022. Over 90% of the patients had stayed on the Red Sea Coast (mostly Hurghada), almost 50% in private residences. Case numbers increased more strongly than traveller numbers. Mosquito control is more difficult in towns than hotel grounds, increasing the risk of infection at private residences. Physicians should consider dengue for unexplained fever after return from Egypt.

German public health authorities noted a strong increase in dengue virus (DENV) infections in persons exposed in Egypt in 2023 (n = 36), especially in the period from September to December (n = 29, data from 23 January 2024). In previous years, never more than eight such infections (median: 3.5 infections) were annually notified in Germany.

We informed our Egyptian colleagues about this observation in December 2023 via the International Health Regulation mechanism. This rapid communication aims to describe the affected travellers and their exposures in order to gain insight into the DENV transmission situation in Egypt.

## German dengue virus surveillance and cases from Egypt in 2023 

Diagnosed DENV infections in Germany are notifiable according to the *Protection against Infection Act* (IfSG) [1]. This includes laboratory-confirmed infections (by single IgM, fourfold IgM/IgG increase, NS1-antigen test or PCR) with at least fever as a symptom (i.e. cases meeting the reference definition), but also laboratory-confirmed asymptomatic infections. A majority of DENV infections are likely to remain asymptomatic; in the others, fever and skin rash are typical symptoms after an incubations period of 3–14 days. The clinical pictures of severe dengue (dengue haemorrhagic fever or dengue shock syndrome) develop rarely. In Germany, deaths from dengue fever among returning travellers is very rare (three deaths in > 10,000 notified cases since 2001).

Although data from 2023 remain incomplete, the overall number of dengue cases from any destinations exceeded 900 and has thus returned to a level seen before the COVID-19 pandemic (2015–2019: 612–1,175 cases/year). Before 2017, notification of DENV infections imported from Egypt was rare (Figure), while from 2017 to 2020, there were between one and eight cases annually; no cases were noted in 2021 and 2022 but in those years, traveller numbers were also lower because of COVID-19-related restrictions. As in 2023, months of onset (or months of diagnosis where onset date was missing) were largely in autumn and winter.

**Figure:**
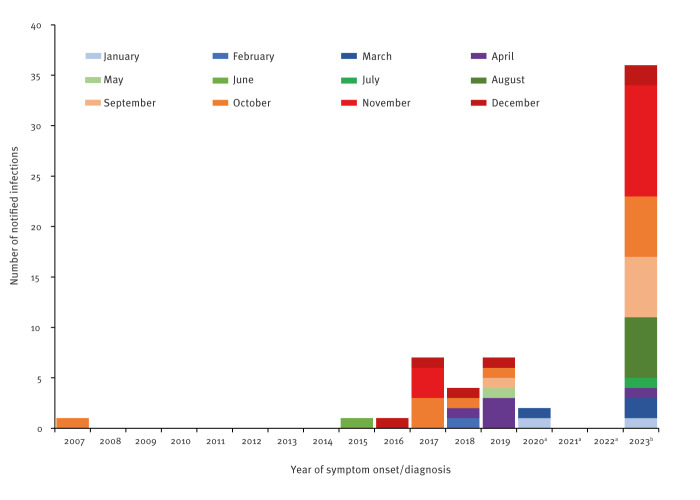
Notified dengue virus infections in travellers returning from Egypt, by month of symptom onset (if unavailable, by month of diagnosis) Germany, 2001–2023 (n = 59)

^a^ Traveller numbers reduced due to the COVID-19 pandemic.

^b^ 2023 data as available on 23 January 2024.

Source: German infectious disease surveillance system: mandatory notifications of laboratory-confirmed infectious disease cases (here dengue cases).

In 2023, 16 men and 20 women aged 11–67 years (median: 36.5 years) were affected. All infections were laboratory-confirmed (three by PCR, 13 by NS1-antigen test, five by IgM/IgG fourfold increase, 15 by single IgM)). Three infections remained asymptomatic, 21 of 36 cases were hospitalised, none died. For 30 cases with complete date information, reported symptom onset was largely in line with exposure in Egypt: 2–24 days (median: 10.5 days) after arrival in Egypt and 17 days before to 15 days after return to Germany (median: 0.5 days after return); calculating from symptom onset. Nine of 30 were probably viraemic while still in Egypt.

## Details on exposures in Egypt

The large majority (30/32) with available information on places of exposure stayed in Red Sea governorate, mostly in the Hurghada region (Table). Among the Hurghada-neighbourhoods, stays were mentioned from El Gouna in the north via the downtown and airport areas to Safaga in the south. One case (symptom onset in October) who had also been to Hurghada suggested having been infected on a brief visit to Qena governorate. We asked for hotel names and received nine geographically widely spaced hotel mentions pertaining to 13 cases (maximum of three cases per hotel). However, 13 of 29 cases with information on type of residence had stayed in private residences in Hurghada (12 cases) or in Cairo (one case).

**Table:** Place of exposures of dengue cases, by type of residence in Egypt, Germany 2023 (n = 32)

Exposure place(s)	Hotel	Private residence	Unclear	Total
Hurghada only	11	10	2	23
Hurghada/Qena/Luxor	0	1	0	1
Hurghada/Cairo/Luxor	1	0	0	1
Hurghada/Cairo/Luxor/Qena	1	0	0	1
El Quseir/Hurghada/Port Ghalib	0	1	0	1
El Quseir only	2	0	0	2
Marsa Alam	0	0	1	1
Cairo only	1	1	0	2
**Total with information**	**16**	**13**	**3**	**32**

Information on place of exposure was available for 32 of the 36 cases. Information on type of residence was available for 29.

Hurghada in this table includes El-Gouna, Makabi Bay, Soma Bay and Safaga, parts of a near continuous tourist area along the coast. El Quseir, Port Ghalib and Marsa Alam are smaller towns/areas with hotels on the Red Sea Coast to the south of Hurghada. Cairo, Luxor and Qena are situated in the Nile River valley.

## Vector presence and dengue virus circulation in Egypt

Dengue fever is caused by infection with one of four dengue viruses (DENV1–4), almost always transmitted by mosquito bites. The container-breeding *Aedes aegypti* (the yellow fever mosquito) is the most typical DENV vector and often found in urban areas. 

Egypt, specifically the Red Sea Coast, was largely free of DENV and *Ae. aegypti,* after the vector’s eradication in the dichlorodiphenyltrichloroethane (DDT) era [2]. But the vector apparently reappeared from the south in the early 2000s [3]. In 2015, the World Health Organization confirmed a dengue fever outbreak in Assiut [4] and in 2017, there was circulation of DENV on the Red Sea Coast affecting travellers [5,6]. Entomological studies confirmed the apparent re-emergence of *Ae. aegypti* in Hurghada and El Quseir in 2017 [7]. The European Centre for Disease Prevention and Control map on *Ae. aegypti* distribution in October 2023 shows presence of the mosquito in Red Sea governorate and parts of the upper Nile valley [8].

Hurghada is the capital of Red Sea governorate. Its services and airport are central to a touristic area stretching at least from El Gouna in the north to Safaga in the south, with most hotel developments directly fronting the Red Sea shore outside of the city. Hurghada only grew from fishing village to city (now 200,000–500,000 inhabitants – estimates vary widely) after the last eradication of *Ae. Aegypti* in the area during the DDT era. 

The entire coast of Red Sea governorate is extremely arid. Hurghada only receives 5.5 mm precipitation per year [9]. There is very little natural land vegetation and there are no bodies of fresh water. 

## Discussion

Egypt is an important year-round travel destination for Germany: In 2022 there were > 150,000 air travellers, among them 70% headed for Hurghada airport. From January to September 2023, the number of travellers was 24% higher than in the same period in 2022 [10], while the number of DENV infections increased more than 10-fold compared with the median of 3.5 cases in the period 2017 to 2022.

We believe the cases notified in Germany are indicators of a noteworthy and increased transmission of DENV on the Egyptian Red Sea Coast, especially in the urban areas like Hurghada city, in 2023. Although single anti-DENV IgM-tests are not very specific for diagnosis of DENV infection, more than half of our notified infections were diagnosed with more specific methods, e.g. PCR or the dengue NS1-antigen assay. Thus, we do not doubt the diagnoses overall. Our observation of DENV infections acquired in Egypt in 2023 fits reports of local outbreaks in Qena governorate (where few international tourists go) and on the Red Sea coast in July 2023 [11]. 

Aside from the increase in absolute numbers, most unusual about the 2023 German cases is the high percentage residing in private residences in towns on the Red Sea. Given the large number of available hotel beds in the region, travellers staying in private residences in the towns seem overrepresented among our cases (data on types of residence of all travellers are not available). This may be a new trend of a larger percentage of tourists staying in accommodations other than hotels, it may indicate a higher risk of DENV infection in those not staying in hotels, or a combination of both.

The hotels have irrigated gardens, probably creating breeding places for *Aedes* mosquitos, whereas gardens and parks are almost absent in the towns in Red Sea governorate. However, achieving effective mosquito control is easier in the hotel gardens than in the towns for a number of reasons: In towns, mosquito breeding sites may be smaller but more geographically spread out and are often situated on private balconies and roofs with limited access for public control efforts. One case anecdotally reported being repeatedly bitten by mosquitos when “cleaning out flowerpots” in their apartment in Hurghada. In addition, wind-exposure at the shore, where the hotels are usually located, may limit the build-up of vector populations more than in the densely built-up areas further inland. And while most affected tourists were not viraemic in Egypt, most cases among local residents probably spend their entire viraemic phase in the towns, so that there are more constant sources of DENV for the vectors in the towns. 

In the German notification data for 2023, more than half of the cases infected in Egypt required hospitalisation, compared with about a third of patients infected in Thailand, a country well-known to be endemic for dengue (p < 0.05; chi-square test). However, this comparison is based on relatively small case numbers for Egypt. The high proportion of hospitalisation in this group suggests a differential underascertainment especially for clinically mild DENV infections acquired in Egypt – perhaps because the country is not recognised by physicians as an area with DENV transmission.

## Conclusion

Dengue virus is currently present on the Red Sea coast and can always be re-introduced by travellers from endemic areas. Transmission of the viruses among inhabitants and tourists can only be reduced or even stopped if the local *Ae. aegypti* populations are reduced or eradicated again. To this end, mosquito control activities probably need to be intensified or started in additional places, especially in the urban areas with *Ae. aegypti* and/or known DENV circulation. As in this arid climate, *Ae.* aegypti breeding grounds are entirely man-made, it is of critical importance to also engage the local population, raising awareness for the need to reduce even small pools of standing water on roofs, in flowerpots etc. Travellers to the region, including those owning or renting holiday residences, ought to actively protect themselves against mosquito bites around the clock. Physicians should consider DENV infection in patients with otherwise unexplained fever returning from Egypt and seek laboratory confirmation, ideally by NS1-antigen test instead of serology. 

In some parts of Germany DENV-competent vectors are increasingly present [12], but autochthonous transmission has not been noted yet. Mosquito control and the prevention of mosquito-borne infections in tourist hotspots with many European travellers is a valuable contribution of destination countries to preventing disease introductions in Europe.
